# Comparison of Automated and Manual DNA Isolation Methods of Liquid-Based Cytology Samples

**DOI:** 10.1089/bio.2018.0148

**Published:** 2019-12-11

**Authors:** Irma G. Domínguez-Vigil, Víctor H. Barajas-Olmos, Lenny Gallardo-Alvarado, Antonio A. Pérez-Maya, Maria L. Garza-Rodríguez, Gerardo I. Magallanes-Garza, Servando Cardona-Huerta, Daniel H. Méndez-Lozano, Oscar Vidal-Gutiérrez, David F. Cantú De León, Hugo A. Barrera-Saldaña

**Affiliations:** ^1^ Departamento de Bioquímica y Medicina Molecular, Facultad de Medicina, Universidad Autónoma de Nuevo León, Monterrey, NL, Mexico.; ^2^ Laboratory for Translational Research, Rudy L. Ruggles Biomedical Research Institute, Western Connecticut Health Network, Danbury, Connecticut.; ^3^ Subdireccion de Investigación Clínica, Instituto Nacional de Cancerología, Ciudad de México, Mexico.; ^4^ Servicio de Oncología, Hospital Universitario “Dr. José Eleuterio González,” Universidad Autónoma de Nuevo León, Monterrey, Nuevo León, Mexico.; ^5^ Tecnologico de Monterrey, Hospital San José, Monterrey, NL, Mexico.; ^6^ Tecnologico de Monterrey, Hospital Zambrano Hellion, San Pedro Garza García, NL, Mexico.; ^7^ Mexican Biobanks Network at Vitagénesis, SA.; ^8^ National Laboratory of Specialized Services of Research, Development, and Innovation for Chemical and Biotechnological Drugs at Innbiogem, SC, Monterrey, Mexico.

**Keywords:** cervical cancer, liquid-based cytology, nucleic acids, quality control, biobank, biomarker

## Abstract

Liquid-based cytology (LBC) has been used as a diagnostic tool for cervical cancer for years and is now being adopted for other gynecological cancers. LBC represents an important challenge to ensure that the process yields representative biospecimens for quality control (QC) of diagnostic procedures. In this study, we compare QC parameters (integrity, yield and purity, and polymerase chain reaction [PCR] amplification) of DNA isolated from LBC (*N* = 296) using two different nucleic acid isolation methods, manual (*n* = 233) or automated (*n* = 63). We also evaluated two different types of cytological brushes for sampling from the cervix. Our results suggest that manual isolation (yield 22.81 ± 1.92 μg) resulted in increased DNA recovery when compared with automated isolation (yield 9.96 ± 1.11 μg) from LBC samples, with a *p*-value of <0.0003. We estimated that 98% (53/54) of the samples preserved the integrity of DNA and were suitable for standard molecular biology analyses. The β-globin gene was amplified in 100% (296/296) of the DNA samples by endpoint PCR. We found no significant difference between the performance of the cytological brushes (*p* value of <0.6711*)* in a general overview. However, when looking at the results from using each brush individually, the manual isolation method was statistically superior to the automated method. Our work illustrates the impact of good QC of preanalytic conditions, which will be important for the application of LBC for developing early detection methods for gynecological cancers.

## Introduction

The Pap smear has been a highly effective screening tool in the diagnosis of cervical cancer (CC)^1^ since its development by George Papanicolaou more than 70 years ago.^2^ More recently, this technique has been modified into a liquid-based Pap smear or liquid-based cytology (LBC) to improve early detection, reduce false-negative rates, and simplify quality control (QC) that is demanded by conventional cervical cytology.^3^ These modifications also offer advantages extending to multiple slide preparation, the incorporation of special stains (immunohistochemistry), human papillomavirus (HPV) detection through DNA testing,^1^ and even the potential to detect other gynecological malignancies, most notably, endometrial and ovarian cancers.^4,5^

QC represents an important challenge in ensuring representative biospecimens for diagnostic purposes and is a prerequisite for performing biomarker discovery.^6,7^ Currently, the scientific community is not focused solely on the final results of investigations based on biospecimens but is more concerned with preanalytical conditions.^8^ QC in both the preanalytical and analytical phases is critical to guarantee reproducible and reliable results. Procedures have been developed to guarantee the quality of conventional biopsies, such as blood (including plasma and serum), saliva, cerebrospinal fluid, synovial fluid, urine, semen, and surgical tissues.^9^

The field of gynecologic oncology is now poised to experience a further diagnostic revolution following the seminal work of Kinde et al., who merged next-generation sequencing (NGS) with LBC for early disease detection.^4^ Since LBC is a noninvasive sampling method with minimal inconvenience for the patients, it offers advantages over more invasive methods, with the overarching potential of using LBC as a preliminary material for biomarker discovery/validation in endometrial and ovarian cancers.

The objective of this study was to compare QC parameters of the DNA extracted and purified from LBC samples obtained using two types of cervical brushes in common use and treated with two different nucleic acid isolation methods.

## Materials and Methods

### Patient enrollment and consent

All women were invited to participate in the research project and an interview was performed. Once the patients agreed to participate, an informed consent form was signed and study enrollment proceeded. All LBC samples were collected over a 2-year period from July 2015 to September 2017 according to protocols approved by the Institutional Ethical and Research Committees of the participants' respective institutes (Facultad de Medicina y Hospital Universitario “Dr. José E. González” [HU] de la Universidad Autónoma de Nuevo Leon [UANL], registration number BI13005; Instituto Nacional de Cancerología [INCan], registration number CEI/1031/16; and San José [HSJ] and Zambrano Hellion [HZH] Hospitals of Tecnologico de Monterrey, registration number 3CEI190390139).

### Sample collection

Cervical sampling was performed by experienced gynecologists and nurses belonging to the staff of their respective institutes using either of two types of brushes available at the time in the clinical settings: (a) Colpotre^®^ (Uileben, Mexico City, Mexico) (*n* = 261), with the cells collected by five clockwise rotations (360°), and (b) Rovers^®^ Cervex-Brush-Combi (Puritan, Guilford, ME) (*n* = 35), with the cells collected by two clockwise rotations (360°). Both preparations of brush-collected cells were immediately transferred into a 50 mL conical tube (CORNING^®^, San Nicolas de los Garza, Nuevo Leon, Mexico) containing 7.5 mL of PreservCyt^®^ solution (ThinPrep^®^; Hologic^®^, Marlborough, MA) for nucleic acid suspension and preservation. All samples were stored at 4°C until processed for nucleic acid isolation within the following 1–52 days. Samples from INCan (Mexico City, Mexico) were transported to the laboratory at the Departmento de Bioquímica y Medicina Molecular at the UANL (Monterrey, Mexico) in a cooler container with gel packs by priority shipping such that delivery occurred within 24 hours (FedEx, Mexico City, Mexico) with an average of 10 LBCs (range 5–15) per shipment.

### DNA isolation

**FIG. 1.**
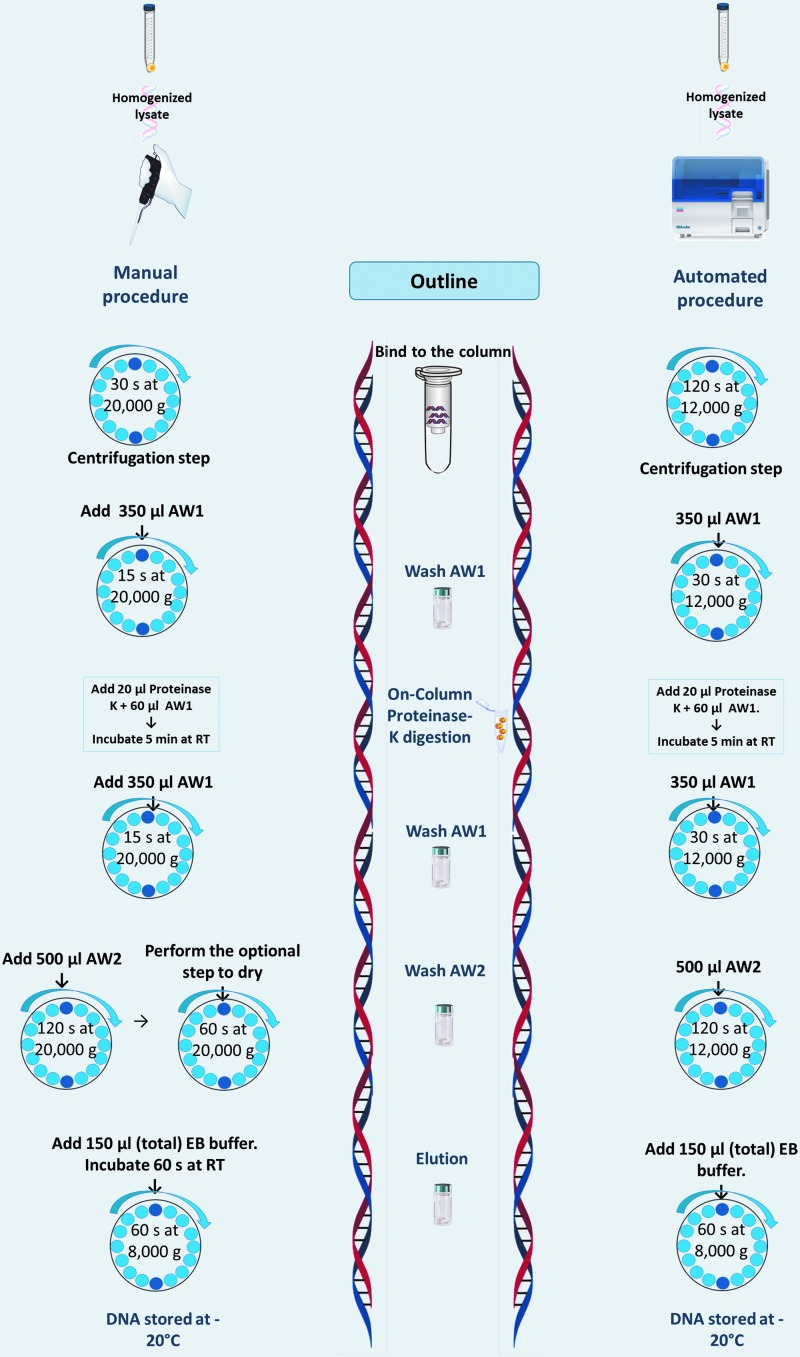
Schematic representation of DNA procedure from LBC samples. *Left side*: manual procedure; *middle*: outline procedure; *right side*: automated procedure. RT = room temperature; EB = elution buffer; AW1 and AW2 = buffer concentrate provided from AllPrep DNA/RNA/miRNA Universal Kit. LBC, liquid-based cytology.

Each sample was vortexed for 30 seconds, centrifuged at 300 *g* for 10 minutes at 4°C, and the supernatant was discarded. The pelleted cells were lysed by adding 600 μL of lysis buffer RLT Plus (Qiagen^®^, Hilden, Germany) (cells were counted to establish the amount of RLT Plus buffer) and homogenized for 30 seconds with a TissueRuptor™ (Qiagen) at medium velocity. Each sample was extracted by either a manual or an automated (Qiacube™; Qiagen, Germantown, MD) method available at the time, following in both cases the protocol of AllPrep DNA/RNA/microRNA Universal Kit™ (Qiagen) as indicated in Figure 1, using 600 μL of homogenized lysate as a starting material. Both manual and automated extractions were performed in the same laboratory and the DNA quantified on the same instrument. Purified nucleic acids were eluted in 100 μL initially, and a second backup elution with an extra 50 μL from the final step of the protocol was biobanked in 1 mL special DNAse and RNAse free cryotubes (Nunc™; Thermo^®^, Waltham, MA). The data from QC were generated from the first 100 μL eluted nucleic acids.

### QC of DNA

DNA was analyzed for integrity, yield, and purity. Suitability of DNA for gene variation analysis was further analyzed by using it as a substrate for amplification of a region of the β-globin gene by endpoint polymerase chain reaction (PCR).

#### Integrity of DNA

The integrity was analyzed by agarose gel electrophoresis. For this purpose, up to 400 ng of DNA, quantified by spectrophotometer, was used and the average size (as an index of integrity) was estimated using a 100 base pairs (bp) DNA ladder (Promega^©^, Fitchburg, WI, and Invitrogen™, Waltham, MA).

#### Measurement of DNA yield and purity

DNA yields were quantified by using a Qubit^®^ 2.0 Fluorometer, with the Qubit™ dsDNA BR Assay Kit for 292 of 296 samples and Qubit dsDNA HS Assay Kit for the rest of the four samples. Purity was judged by the A_260nm_/A_280nm_ and A_260nm_/A_230nm_ absorbance ratios by using a UV-Vis NanoDrop™ 2000 spectrophotometer (Thermo).

#### PCR amplification as a functional assay of DNA quality

The β-globin gene (*HBB*) was amplified using GoTaq Colorless Master Mix (Promega), following the manufacturer's instructions. The reaction mixture of 0.2 μM for each of the β-globin gene primers (forward: 5′-CAACTTCATCCACGTTCACC-3′ and reverse: 5′-GAAGAGCCAAGGACAGGTAC-3′; IDT, Skokie, IL), 100 ng of DNA quantified by spectrophotometer (seven samples contained less than 100 ng), and GoTaq Colorless Master Mix. The reaction volume was adjusted with Milli-Q water to a final volume of 13.5 μL. After an initial denaturation step at 94°C for 5 minutes, amplification was carried out for 30 cycles, comprising each of the following successive steps of incubation at 94°C (denaturation) for 30 seconds, incubation (annealing) at 57°C for 30 seconds, and at 72°C (elongation) for 2 minutes. Cycling concluded with a final extension step at 72°C for 10 minutes. Amplification was performed using a Veriti Thermal Cycler (Applied Biosystems^®^, Foster City, CA). The expected prominent PCR product of 268 bp was confirmed following electrophoresis of amplification product in 2% agarose gel.

### Statistical analysis

Considering the variables of brush types, isolation protocols used, and storage time before processing/nucleic acid isolation, we compared the yield based on fluorescence of dsDNA preparations. Quantitative results obtained were analyzed using the GraphPad Prism 5 Software package (GraphPad Software, Inc., La Jolla, CA). Data are presented as the mean ± standard error of the mean. Unpaired data were compared using the unpaired Student's *t*-test. A *p*-value of <0.05 was considered significant. One-way analysis of variance was used to compare the yield of nucleic acids versus time until the isolation process.

## Results

Two hundred ninety-six women were enrolled for this study, with a mean age of 49 (±13 standard deviation) years and range of 19–85 years. Sixty-one LBCs from participants were collected and processed within 24 hours. Two hundred thirty-five biospecimens from study participants at the other centers were collected, stored at 4°C for transport to our processing center. DNA from the samples was isolated by either manual (*n* = 233) or automated (*n* = 63) procedure as indicated in Figure 1. Samples were assigned to either processing procedure available at the time. Ninety-six percent (226/235) of samples from out of town were processed between 1 and 35 days. Four samples were isolated at later time points, between 36 and 52 days.

### The integrity of DNA is suitable for standard molecular biology analyses

**FIG. 2.**
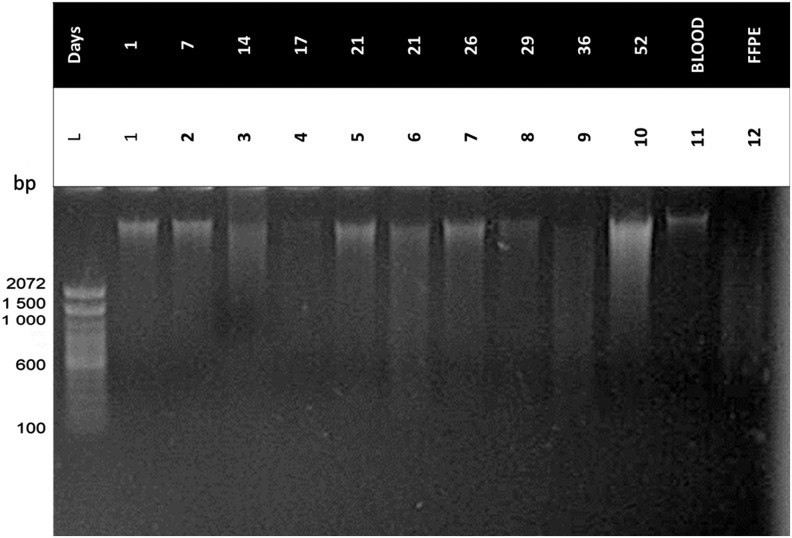
Integrity assessment of isolated nucleic acids. Lane L: DNA ladder 100 bp; Lanes 1–10: DNA of LBC samples taken by Colpotre^®^ and isolated by manual method; Lane 11: DNA of blood sample; Lane 12: DNA from formalin-fixed paraffin-embedded tissue; Conditions: 400 ng of DNA in agarose gel at 1% at 110 volts during 60 minutes. *First row* indicates sample storage time (days) before isolate DNA.

DNA integrity was investigated by electrophoresis in 1% agarose gel and was judged by the proportion of high- versus low-molecular-weight DNA as suggested by evidence of smearing (Fig. 2). Based on this qualitative assessment, it was estimated that 98% (53/54) of the samples tested were of sufficient quality.

### DNA yield from LBC samples is more efficient by manual isolation

**FIG. 3.**
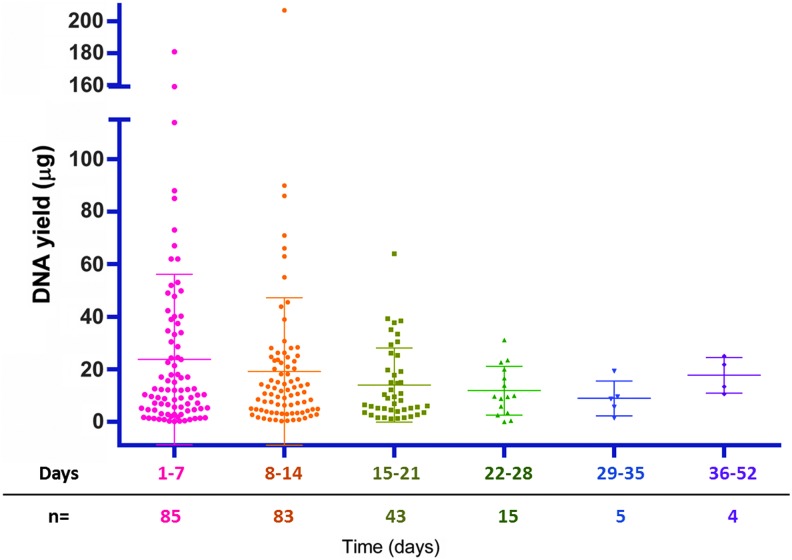
Effect of storage time before isolation of DNA from LBC samples. No significant differences were observed (*p* < 0.3128).

The efficiency of DNA extraction was compared between the two isolation methods and the type of brushes (Rovers vs. Colpotre) used by evaluating their impact on the QC parameters of the characterized samples (Table 1). In this study, the manual isolation method resulted in increased DNA recovery when compared with the automated isolation method (*p* < 0.0003***), regardless of the type of brush used: Colpotre (*p* < 0.0022**) or Rovers (*p* < 0.0445*). Additionally, the variable of time between sample collection and processing was analyzed to determine if it impacted the nucleic acid yield from the PreservCyt solution-stored cells (Fig. 3). Most of the INCan samples, 71% (168/235), were isolated within the 1–14 days after collection; the effect of the time in these samples' yield was not significant (*p* < 0.3128).

**Table 1.** DNA Yield from Liquid-Based Cytology Samples

(a) Type of isolation: manual vs. automated	Manual (n = 233)	Automated (n = 63)	p-*Value*
DNA yield (μg)	22.81 ± 1.92	9.96 ± 1.11	<0.0003^***^
Purity 260/280	1.83 ± 0.02	1.81 ± 0.02	<0.6144
Purity 260/230	1.29 ± 0.04	1.41 ± 0.09	<0.2219

(b) Type of brush: Colpotre^®^ vs. Rovers^®^	Colpotre (n = 261)	Rovers (n = 35)	p-*Value*
DNA yield (μg)	20.11 ± 1.67	18.04 ± 4.55	<0.6711
Purity 260/280	1.83 ± 0.02	1.81 ± 0.03	<0.7181
Purity 260/230	1.32 ± 0.04	1.26 ± 0.11	<0.6353

(c) Colpotre brush: manual vs. automated	Manual (n = 209)	Automated (n = 52)	p-*Value*
DNA yield (μg)	22.75 ± 2.03	9.88 ± 1.28	<0.0022^**^

(d) Rovers brush: manual vs. automated	Manual (n = 24)	Automated (n = 11)	p-*Value*
DNA yield (μg)	24.18 ± 6.25	4.62 ± 1.37	<0.0445^*^

(e) Type of isolation and brushes	Manual (n = 233)			Automated (n = 63)
	Colpotre (n = 209)	Rovers (n = 24)	p value	Colpotre (n = 52)	Rovers (n = 11)	p-*Value*
DNA yield (μg)	22.65 ± 2.03	24.18 ± 6.25	<0.8094	9.88 ± 1.28	4.62 ± 1.37	<0.0735
Purity 260/280	1.83 ± 0.02	1.83 ± 0.02	<0.9709	1.82 ± 0.02	1.76 ± 0.05	<0.4083
Purity 260/230	1.29 ± 0.04	1.31 ± 0.14	<0.8646	1.46 ± 0.10	1.15 ± 0.17	<0.2232

Data are presented as the MEAN ± SEM. ^*^p ≤ 0.05, ^**^p ≤ 0.01, and ^***^p ≤ 0.001 values (bold) were considered statistically significant. The yield was generated from the first 100 μl eluted nucleic acids.

### Amplifiability of DNA isolated from LBC samples

**FIG. 4.**
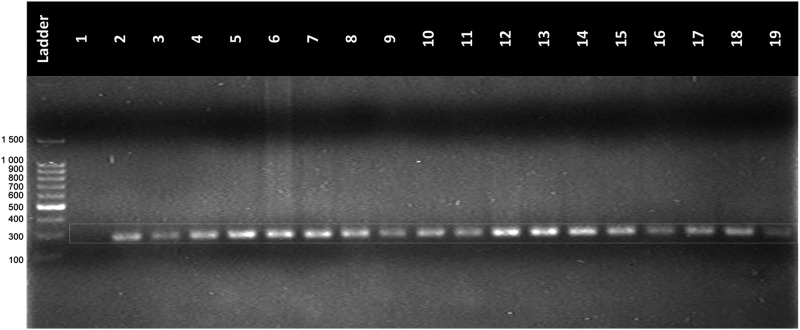
Amplification of β-globin gene region from DNA isolated from LBC samples. Lane L: DNA ladder; Lane 1: negative control; Lanes 2–18: amplicon DNA of LBC samples; Lane 19: positive control amplicon DNAg previously known; Conditions: 8 μL of amplicon DNA in agarose gel at 2% at 95 volts during 90 minutes.

The 268 bp band of the β-globin gene was amplified in 100% (296/296) of the DNA samples by endpoint PCR (Fig. 4).

## Discussion

In this study, two DNA isolation methods from LBC samples were tested to compare their efficacies to yield good quality and quantity of DNA. Results demonstrated that the manual isolation of DNA from LBC is more effective than automated isolation under those conditions.

Initial handling and proper conservation of valuable DNA samples represent critical steps for the efficient utilization of LBC samples as the starting material for biomarker discovery.^10^ Biobanking and biospecimen science studies have emerged as a global priority in view of the critical importance of preanalytical precautions and their relevance in omics research for biomarker discovery.^11^ Nevertheless, studies addressing the QC of LBC samples are limited.^12–17^

Boulet et al. in 2008 demonstrated that the recovery of nucleic acids from cytology was possible even when a cytology sample had been stored for up to 10 years (archival smears), with all samples providing DNA suitable for PCR amplification with amplicons up to 400 bp.^14^ These studies demonstrated that long-term stored LBC samples are stable for the isolation of these nucleic acids. In this study, we chose a short amplicon; however, a long-range PCR should be designed for those downstream applications where less degraded DNA is required.

Serrano et al.^18^ isolated DNA, RNA, and proteins from LBC resuspended in saline solution buffer and concluded that preservation solutions (PAXgene™ or PreservCyt) can influence integrity and yield. In addition, the suitability of archived LBC cytological samples for molecular biology analyses has been investigated. In a study of 8-year-old samples stored at room temperature in PreservCyt solution, degradation of DNA by analysis of β-globin amplification and cytological nuclear preservation features were time-dependent, declining with increasing storage time.^19^ In this study, we demonstrated that storage time of LBC samples in PreservCyt for several weeks is safe at 4°C. This methanol-based preservation solution is intended for ThinPrep Pap™ testing, even if kept between 15°C (59°F) and 30°C (86°F) and for up to 6 weeks.^20^ Notably, PreservCyt is used in routine clinical practice with the Hybrid Capture 2 (HC2) for CC screening, and it is also provided by Mexico's National Health Social Security System.^21^

The nucleic acid degradation evidenced by the smears in the gel lanes in Figure 2 can be explained by autolysis that occurs during normal epithelial cell maturation and changes that occurred after exfoliation.^12,15,22^

More recently, LBC studies have focused on differences between sampling devices, such as Cervex Brush^®^ versus Cervex Brush^®^ Combi, without having identified differences in DNA yield.^23^ Similarly, in this study, we did not identify significant differences in overall yield between two different brush types (comparison for DNA yield *p* < 0.6711; Table 1, section b); significance was established when the manual versus automated DNA isolation from an LBC using the Colpotre (*p* < 0.0022**; Table 1, section c) and the Rovers (*p* < 0.0445; Table 1, section d). Colpotre is a Mexican-made brush, similar to the Cervex Brush, but is approximately 10 times less expensive than the Rovers Cervex Brush (retail cost in Mexico 0.25 USD and 2.57 USD, respectively). Therefore, according to this study, we believe that the two brushes were equally effective with regard to DNA yields, despite the differences in price.

To reduce operator-induced variations and more readily standardize throughput, we also tested a benchtop automated sample preparation machine (Qiacube; Qiagen), a low-throughput instrument that processes up to 12 samples per run. Automated protocols can be an alternative for more standardized, reproducible, and efficient protocols designated especially for high-throughput analyses, reducing hands-on time.

As illustrated in Figure 1, the standard protocol for the automated method does not include the optional centrifugation spin to dry the column and prevent the carryover of ethanol in the DNA elution step; moreover, we did not find a statistically significant difference between manual and automated methods with respect to DNA purity. Furthermore, in the automated protocol, there is no incubation time before the DNA elution, which can result in low nucleic acid yield because DNA is still bound to the column membrane.^24^ Additionally, the centrifugation speed on the Qiacube automated method was only 12,000 *g*, whereas in the manual method, centrifugation was performed at 20,000 *g*. Even though the incubation step could not be performed by the automated method, a considerable amount of DNA was obtained. It is important to mention if the user needs a specific protocol for the automated method, the company can design it based on the user's requirements.

In this study, and as judged by DNA yield measurements, manual isolation resulted in greater yields, and this finding is in agreement with previous reports.^25,26^ However, the difference in yields may be attributed to the larger sample size for the manual method versus the automated method. Even if this method was superior, the advantages of the automation cannot be ignored and the choice of the DNA isolation method should be based on the workflow of each biobank.

Spectrophotometry is a traditional and economically convenient method of quantifying DNA in molecular biology laboratories due to its accessibility and also in biobank studies.^27^ However, other methods such as fluorometry by Qubit™, have proven more reliable due to the specificity for double-stranded DNA. All the yield comparisons shown in Table 1 were performed based on fluorometry, and all the purity comparisons were performed based on spectrophotometry.

In this study, we obtained average DNA yields of 22.81 μg (±1.92) and 9.96 μg (±1.11) using the manual and automated isolation methods, respectively, from an LBC sample, and reasonable integrity of DNA was verified, as seen in the prominence of a high molecular band (Fig. 2). Most traditional clinical molecular pathology assays are designed considering the degradation of DNA, rendering fragments of lengths varying between 100 and 400 bp, and typically require less than 50 ng per analysis. However, the new technologies use significantly less DNA. In this study, we were able to obtain a considerable amount of DNA with both isolation methods.

Other general considerations on the isolation of nucleic acids are the dependent variables of the patient, such as the phase of her menstrual cycle, and those on the nurse/doctor, such as her/his skill to collect the sample.

The primary responsibility of biobanks is to collect biospecimens that truly represents features of the local population.^28^ CC is the fourth leading cause of cancer death in women, with 311,000 worldwide deaths, and is the second leading cause of cancer death in women in Mexico with 4,121 deaths.^29^ Europe has a Cervical Cytology Biobank, which is a resource for molecular epidemiology and for evaluating CC screening and intervention approaches.^16,28,30^ Currently, LBC is used for CC screening. When LBC is used in combination with NGS technology, it can be beneficial in developing early detection methods in other gynecological cancers. Since there are no early detection biomarkers currently known that are specific enough to detect ovarian and endometrial cancers, these two cancers have started to gain more attention given their lethality.

For these reasons, our work represents a guide to QC for collecting LBC samples and stresses the impact of good control of preanalytical conditions while obtaining samples (i.e., LBCs) in clinics far from a laboratory, to favor their quality as starting material for biomarkers discovery in the fight against the feared gynecological cancers.
